# Brain drain attitudes and life satisfaction among dental students in Türkiye: a multidimensional analysis

**DOI:** 10.1186/s12909-026-09695-6

**Published:** 2026-06-24

**Authors:** Fatih Karaaslan, İlayda Kavak

**Affiliations:** Department of Periodontology, Faculty of Dentistry, Uşak University, Uşak, Türkiye

**Keywords:** Migration attitudes, Dental education, Subjective well-being, Workforce planning, Career intentions, Health professional migration

## Abstract

**Background:**

Migration-related attitudes among healthcare trainees have become an important concern for workforce sustainability. However, the psychosocial and contextual correlates of these attitudes among dental students remain underexplored. This study aimed to assess attitudes toward brain drain among dental students in Türkiye and to examine their associations with life satisfaction, sociodemographic characteristics, and family background factors.

**Methods:**

This cross-sectional study included 935 undergraduate dental students recruited through national student networks across multiple dental faculties in Türkiye. Data were collected using an anonymous online questionnaire comprising a demographic information form, the Life Satisfaction Scale (LSS), and the Attitude Scale for Brain Drain (ASBD). Statistical analyses included non-parametric group comparisons, Spearman’s correlation analyses, hierarchical multiple linear regression, and supplementary sensitivity analyses.

**Results:**

A substantial proportion of students (61.9%) reported an intention to work abroad after graduation. Brain drain attitudes were accompanied by strong family support for working abroad (70.9%) and a high desire to return after migration (67.1%). Total, social, and economic life satisfaction showed statistically significant negative correlations with ASBD scores, with the largest correlation observed for economic life satisfaction (rho = -0.291, *p* < .001). In the hierarchical regression model, total life satisfaction (β = -0.248, *p* < .001), academic year, age, and maternal education remained independently associated with ASBD scores.

**Conclusions:**

Brain drain attitudes were common among participating dental students in Türkiye and were associated with life satisfaction, academic year, age, and family background factors. These findings suggest that migration-related attitudes are shaped not only by personal well-being but also by broader educational, familial, professional, and structural influences.

**Supplementary Information:**

The online version contains supplementary material available at 10.1186/s12909-026-09695-6.

## Introduction

Brain drain, commonly defined as the migration of highly skilled individuals to countries offering more favorable professional, economic, and social conditions, remains a major challenge for health systems worldwide because it weakens workforce capacity and undermines the return on investment in professional training [1]. In the health sector, migration-related decisions are shaped by interacting push and pull factors, including income, working conditions, occupational safety, career opportunities, and quality of life [2]. Importantly, such attitudes may begin to develop before graduation, as students start to evaluate their future professional prospects and social position within the health system [3].

Within this broader framework, dentistry warrants particular attention. Dental education is lengthy, costly, and highly dependent on institutional resources and supervised clinical training [4]. The migration of newly trained dentists may therefore affect not only individual career pathways but also workforce planning, service accessibility, and the return on educational investment [5]. In Türkiye, this issue appears to be shaped by a strained structural context characterized by the rapid expansion of dental faculties and student quotas, limited employment opportunities, and concerns about professional sustainability [6–8]. National and professional data further suggest a mismatch between training expansion, labor-market absorption, and service utilization in oral healthcare [6–9].

Recent studies from Türkiye and other countries indicate that migration-related attitudes are common among health professional students, including those in medicine, nursing, and dentistry [10–12]. These attitudes appear to be shaped by multiple interacting influences, including family and social environments, psychosocial pressures, career goals, and broader opportunity structures [12–14]. This suggests that migration-related attitudes should be understood as multidimensional rather than as the product of a single explanatory factor. Among the individual-level factors potentially relevant to these attitudes, life satisfaction is especially important. Life satisfaction refers to a person’s cognitive evaluation of overall life quality in relation to personal expectations [15, 16]. In university students, it is shaped by economic, academic, and social experiences, and in dental education it may be particularly influenced by financial pressures, academic workload, clinical demands, and uncertainty about future employment. In the Turkish context, psychometric evidence also distinguishes between economic and social dimensions of life satisfaction, suggesting that these domains may not contribute equally to broader evaluations of well-being [17].

Despite increasing concern about outward migration in Turkish healthcare, evidence specifically focusing on dental students in Türkiye remains limited. Previous studies have examined migration intentions or brain drain attitudes among health professional students, including medical, nursing, and dental students, and some have explored related psychosocial factors such as life satisfaction, future anxiety, or career stress [10–12, 18, 21, 22, 26]. However, research integrating brain drain attitudes, life satisfaction dimensions, and sociodemographic and family background factors among dental students in Türkiye within a single analytical framework remains scarce. This represents an important gap, as dental students are still in training yet are already forming professional expectations within a context marked by expanding educational capacity, workforce pressures, and concerns about long-term professional sustainability. Therefore, this study aimed to assess attitudes toward brain drain among dental students in Türkiye and to examine their associations with life satisfaction, sociodemographic characteristics, and family background factors. The study was guided by the following hypotheses:

- H1: Lower life satisfaction would be associated with stronger brain drain attitudes.
- H2: The economic dimension of life satisfaction exerts a more pronounced influence on brain drain attitudes than the social dimension.
- H3: Academic year and family-related socioeconomic characteristics would show stronger associations with brain drain attitudes than basic demographic factors such as gender.

## Methods

### Study design and ethical considerations

This cross-sectional, web-based survey study was conducted among undergraduate dental students in Türkiye between September 2024 and March 2025. The study was coordinated by the Faculty of Dentistry at Uşak University and reported in accordance with the Checklist for Reporting Results of Internet E-Surveys (CHERRIES) [19]. Ethical approval was obtained from the Uşak University Faculty of Medicine Non-Interventional Clinical Research Ethics Committee (Approval No: 621-621-19), and the study was conducted in accordance with the Declaration of Helsinki.

Before accessing the questionnaire, participants were informed about the study purpose, voluntary participation, anonymity, and confidentiality. Electronic informed consent was obtained from all participants. No personally identifiable information, such as names, student numbers, or IP addresses, was collected, and all responses were analyzed in aggregate form.

### Participants and data collection

The target population comprised undergraduate dental students enrolled in years 1–5 at dental faculties in Türkiye. Students were eligible to participate if they were currently enrolled in a dental school in Türkiye, provided electronic informed consent, and completed the online questionnaire. Data were collected using an anonymous, self-administered Google Forms survey.

The survey link was disseminated nationally through broad student networks, including WhatsApp student groups, student representatives, student branches, and professional student associations related to dentistry. Through these networks, the survey was circulated to students from multiple dental faculties across Türkiye. Recruitment was based on non-probability convenience sampling, as participation depended on access to the survey link and willingness to respond.

### Sample size and power analysis

An a priori power analysis was performed using G*Power version 3.1.9.7 to estimate the minimum sample size required for hierarchical multiple linear regression. Based on a small effect size (Cohen’s f² = 0.025), α = 0.05, and 85% power, the minimum required sample size was calculated as 846 participants. The final sample size of 935 participants exceeded this requirement and was therefore considered adequate for the planned analyses.

### Measures

#### Life Satisfaction Scale (LSS)

Life satisfaction was assessed using the Life Satisfaction Scale (LSS), a psychometrically validated instrument developed for the Turkish population by Köse et al. [17]. The LSS consists of 8 items rated on a 5-point Likert scale and includes two subdimensions: Social Life Satisfaction (SLS) and Economic Life Satisfaction (ELS). Total scores range from 8 to 40, with higher scores indicating greater life satisfaction. In the present sample, reliability coefficients calculated using available subscale scores indicated acceptable consistency for the LSS (alpha = 0.753; Spearman-Brown coefficient = 0.754).

#### Descriptive information form

The descriptive information form collected sociodemographic, socioeconomic, and migration-related variables relevant to the study objectives. Variables included gender, age, academic year, maternal and paternal education levels, and family monthly income. Age was recorded using nine ordinal categories ranging from 18 to ≥ 26 years to support participant anonymity and reflect the distribution of the dental student cohort. Academic year was classified from 1st to 5th year, and parental education was categorized from primary school to university level. Family monthly income was classified into low, middle, and high income categories based on national minimum wage benchmarks at the time of data collection.

Six binary items (Yes/No) were used to assess migration-related and professional perceptions, including intention to work abroad after graduation, family support for working abroad, feeling valued as a dental student in Türkiye, optimism about the future of the profession, desire to return after migration, and feeling indebted to the country.

#### Attitude Scale for Brain Drain (ASBD)

Attitudes toward brain drain were measured using the Attitude Scale for Brain Drain (ASBD), a validated 16-item instrument developed by Öncü et al. [20]. The ASBD includes 14 positively phrased items and 2 reverse-coded items, all rated on a 5-point Likert scale. Total scores range from 16 to 80, with higher scores indicating stronger attitudes toward brain drain. The scale includes two subdimensions: Pull Factors (PL) and Push Factors (PS). In the present sample, reliability coefficients calculated using available subscale scores indicated moderate alpha reliability and a high Spearman-Brown coefficient for the ASBD (alpha = 0.665; Spearman-Brown coefficient = 0.847).

#### Statistical analysis

All statistical analyses were performed using IBM SPSS Statistics for Windows, version 26.0 (IBM Corp., Armonk, NY, USA). Continuous variables were summarized as mean ± standard deviation (M ± SD), and categorical variables were summarized as frequencies and percentages. The distribution of scale scores was assessed before selecting the appropriate statistical tests.

Because scale scores did not meet normality assumptions, non-parametric tests were used for group comparisons. Chi-square tests were used to examine differences in categorical migration-related variables according to academic year, gender, parental education, and family monthly income, with Cramér’s V reported as the effect size. Kruskal-Wallis tests were used to compare LSS, ELS, SLS, ASBD, PS, and PL scores across academic year, parental education, and family monthly income. When significant, post hoc comparisons were performed using Bonferroni-adjusted Dunn tests, and epsilon-squared (ε²) was reported as the effect size. Gender differences in scale scores were evaluated using the Mann-Whitney U test, with effect sizes expressed as r.

Spearman’s rank correlation coefficients were calculated to examine associations between life satisfaction scores (ELS, SLS, and total LSS) and brain drain attitude scores (PS, PL, and total ASBD). Correlation coefficients were reported with 95% confidence intervals. Reliability coefficients were calculated using available subscale scores and reported using alpha and Spearman-Brown coefficients.

To examine independent associations with brain drain attitudes, hierarchical multiple linear regression was performed using the total ASBD score as the dependent variable. Demographic variables were entered in the first block, family background variables in the second block, and total LSS score in the final block. Although group comparisons were conducted using non-parametric methods due to non-normal score distributions, regression analysis was used to evaluate the independent associations of selected variables after assessing model assumptions at the residual level. Categorical predictors were entered using reference categories.

Age was additionally examined as a continuous variable in a sensitivity analysis. Results from this model were compared with the categorical age model and are reported in the Supplementary Material. The hypothesis that economic life satisfaction has a stronger association with brain drain attitudes than social life satisfaction was formally tested using Williams’ test for dependent correlations. To complement the linear regression model, intention to work abroad was additionally modeled using logistic regression, with results reported as odds ratios (ORs) and 95% confidence intervals in the Supplementary Material.

Model assumptions were assessed by examining multicollinearity using variance inflation factor (VIF) values, with values below 5 considered acceptable. Heteroscedasticity was evaluated using the Breusch-Pagan test. Model performance was evaluated using R², adjusted R², ΔR², and F-change statistics, and regression coefficients were reported as B, SE, β, t, and p values. A two-sided p value < 0.05 was considered statistically significant for all analyses.

## Results

A total of 942 responses were received. After screening for eligibility and completeness, 935 questionnaires (99.3%) were included in the final analysis; seven responses (0.7%) were excluded because they were incomplete or did not meet the eligibility criteria. Because the survey was distributed through open online channels, the total number of students who received or viewed the invitation could not be determined; therefore, a conventional response rate could not be calculated. In addition, as no personally identifiable information or IP addresses were collected, duplicate submissions could not be formally verified. Among the 935 undergraduate dental students included in the analysis, most participants were female, the largest academic group was 4th-year students, the most common age was 22 years, and most participants reported a family monthly income below 60,000 TL (Table 1). Reliability coefficients for the study scales are presented in Table 2.

**Table 1:** Sociodemographic Characteristics of the Participating Dental Students (*N* = 935)

Variable	Category	*n* (%)
Gender		
	Female	599 (64.1%)
	Male	336 (35.9%)
Age (years)		
	18	68 (7.3%)
	19	125 (13.4%)
	20	125 (13.4%)
	21	158 (16.9%)
	22	188 (20.1%)
	23	152 (16.3%)
	24	73 (7.8%)
	25	27 (2.9%)
	≥ 26	19 (2.0%)
Academic Year		
	1st year	166 (17.8%)
	2nd year	140 (15.0%)
	3rd year	188 (20.1%)
	4th year	294 (31.4%)
	5th year	147 (15.7%)
Mother’s Education*		
	Primary school	299 (32.0%)
	Secondary school	124 (13.3%)
	High school	245 (26.2%)
	University	267 (28.6%)
Father’s Education*		
	Primary school	152 (16.3%)
	Secondary school	115 (12.3%)
	High school	238 (25.5%)
	University	430 (46.0%)
Family Monthly Income (TL)		
	< 60,000 TL	608 (65.0%)
	60,000–120,000 TL	281 (30.1%)
	> 120,000 TL	46 (4.9%)

*: Educational categories were reported based on participants’ self-reports and represent the Turkish education system

**Table 2:** Reliability Coefficients for the Study Scales

Scale	Components	Raw α	Spearman-Brown	*N* items	95% CI
LSS	ELS + SLS	0.753	0.754	2 subscales	[0.720, 0.783]
ASBD	PS + PL	0.665	0.847	2 subscales	[0.619, 0.706]

Reliability estimates were derived from subscale total scores because item-level data were unavailable. For these two-component scales, the Spearman-Brown coefficient was also provided as a standardized reliability estimate. 95% CI = 95% confidence interval. *LSS *Life Satisfaction Scale, *ASBD *Attitude Scale for Brain Drain, *ELS *Economic Life Satisfaction, *SLS *Social Life Satisfaction, *PS *Push Factor, *PL *Pull Factor

Academic year was significantly associated with all six migration-related variables, with small effect sizes (Cramér’s V range: 0.114–0.211). The intention to work abroad was highest among 2nd-year students and lower among 4th- and 5th-year students. Feelings of being valued as a dental student and optimism about the future of the profession were generally lower in later academic years. Gender differences were limited; only feeling indebted to the country differed significantly by gender, with female students reporting higher indebtedness than male students (Table 3).

**Table 3:** Academic Year and Gender Differences in Migration-Related Variables (Chi-Square Tests with Effect Sizes)

Item (% Yes)	Academic Year									Gender				
	Total	I	II	III	IV	V	χ²(4)	*p*	Cramér’s V	Male	Female	χ²(1)	*p*	Cramér’s V
Item I	61.9	66.3	76.4	64.9	54.4	54.4	25.049	< 0.001*	0.164	59.2	63.4	1.447	0.229	0.039
Item II	70.9	74.7	82.9	69.1	66.7	66.0	15.419	0.004*	0.128	70.5	71.1	0.013	0.910	0.004
Item III	33.0	51.8	38.6	31.4	24.8	25.2	41.664	< 0.001*	0.211	33.6	32.7	0.045	0.833	0.007
Item IV	29.9	36.1	38.6	27.7	25.5	26.5	12.049	0.017*	0.114	32.7	28.4	1.746	0.186	0.043
Item V	67.1	77.7	67.1	63.8	68.7	55.8	18.238	0.001*	0.140	63.4	69.1	2.937	0.087	0.056
Item VI	52.0	66.9	53.6	56.9	43.5	44.2	28.660	< 0.001*	0.175	47.0	54.8	4.853	0.028*	0.072

Percentages represent “Yes” responses. Academic year comparisons were analyzed using chi-square tests with 4 degrees of freedom, while gender comparisons were analyzed with chi-square tests with 1 degree of freedom. Cramér’s V indicates the effect size. *p* < .05 denotes statistical significance. Items include Item I (intention to work abroad after graduation), Item II (family support for working abroad), Item III (feeling valued as a dental student in Türkiye), Item IV (optimism about the future of the profession), Item V (desire to return after migration), and Item VI (feeling indebted to the country)

Maternal education was associated with several migration-related variables, including intention to work abroad, family support for working abroad, desire to return after migration, and feeling indebted to the country. Students whose mothers had university education reported higher intention to work abroad and greater family support for working abroad than those whose mothers had primary school education. In contrast, desire to return after migration and feeling indebted to the country were higher among students whose mothers had lower education levels. Paternal education showed a more limited pattern: higher paternal education was mainly associated with greater family support for working abroad, whereas desire to return and feelings of indebtedness were higher among students whose fathers had lower education levels. Overall, effect sizes were small, indicating that parental education was associated with migration-related perceptions but explained only limited variation (Table 4).

**Table 4:** Parental Education Differences in Migration-Related Variables (Chi-Square Tests with Effect Sizes)

Item (% Yes)	Mother’s Education Level							Father’s Education Level						
	Primary school	Secondary school	High school	University	χ²(3)	*p*	Cramér’s V	Primary school	Secondary school	High school	University	χ²(3)	*p*	Cramér’s V
Item I	55.5	63.7	61.6	68.5	10.336	0.016*	0.105	59.9	60.9	63.4	62.1	0.565	0.904	0.025
Item II	59.2	73.4	73.5	80.5	32.996	< 0.001*	0.188	59.2	61.7	73.1	76.3	21.342	< 0.001*	0.151
Item III	32.1	29.0	35.9	33.3	1.946	0.584	0.046	36.2	33.0	29.8	33.7	1.876	0.598	0.045
Item IV	27.4	36.3	29.4	30.3	3.341	0.342	0.060	30.9	27.8	34.5	27.7	3.678	0.298	0.063
Item V	73.9	67.7	66.1	59.9	12.634	0.005*	0.116	82.2	75.7	60.1	63.3	27.753	< 0.001*	0.172
Item VI	59.5	51.6	46.1	49.1	11.116	0.011*	0.109	66.4	53.0	43.7	51.2	19.454	< 0.001*	0.144

Percentages show responses of “Yes.” Comparisons based on mothers’ and fathers’ education levels were analyzed using chi-square tests with 3 degrees of freedom. Cramér’s V indicates the effect size. *p* < .05 signifies statistical significance. Item I (intention to work abroad after graduation), Item II (family support for working abroad), Item III (feeling valued as a dental student in Türkiye), Item IV (optimism about the future of the profession), Item V (desire to return after migration), and Item VI (feeling indebted to the country)

Family monthly income was associated with family support for working abroad and desire to return after migration. Students in the high-income group reported the highest family support for working abroad, whereas desire to return after migration was higher among students in the low-income group. These associations also showed small effect sizes, while other migration-related variables did not show meaningful income-based differences (Table 5).

**Table 5:** Migration-Related Variables by Family Monthly Income (Chi-Square Tests with Effect Sizes)

Item (% Yes)	Low	Middle	High	*χ²(2)*	*p*	Cramér’s V
Item I	61.8	60.5	71.7	2.124	.346	.048
Item II	66.9	75.8	93.5	19.260	<.001*	.144
Item III	32.9	34.5	26.1	1.289	.525	.037
Item IV	29.9	29.5	32.6	0.178	.915	.014
Item V	70.1	62.3	56.5	7.709	.021*	.091
Item VI	53.8	49.1	45.7	2.457	.293	.051

Percentages indicate “Yes” responses. Comparisons by family monthly income were analyzed using chisquare tests with 2 degrees of freedom. Cramér’s V measures effect size. *p* < .05 signifies statistical significance. Item I (intention to work abroad after graduation), Item II (family support for working abroad), Item III (feeling valued as a dental student in Türkiye), Item IV (optimism about the future of the profession), Item V (desire to return after migration), and Item VI (feeling indebted to the country)

Academic year was significantly associated with all life satisfaction and brain drain scale scores. First-year students had the highest economic and total life satisfaction scores, whereas 2nd-year students had the highest push, pull, and total ASBD scores. Effect sizes were small, with the largest academic-year effect observed for economic life satisfaction (ε² = 0.040). Gender differences in scale scores were generally not statistically significant, indicating that scale-based patterns were more strongly related to academic year than to gender (Table 6).

**Table 6:** Scale Scores by Academic Year and Gender (Kruskal-Wallis and Mann-Whitney U Tests with Effect Sizes)

Scale	Academic Year									Gender				
	Total	I	II	III	IV	V	H(4)	*p*	ε²	Male	Female	U	*p*	*r*
ELS	11.97 ± 2.94	13.19 ± 2.45	11.99 ± 2.93	11.85 ± 2.86	11.54 ± 2.88	11.57 ± 3.32	40.950	< 0.001	0.040	11.99 ± 3.02	11.95 ± 2.89	100,937	0.938	0.003
SLS	14.24 ± 3.07	14.92 ± 2.59	14.36 ± 2.73	14.17 ± 3.07	13.96 ± 3.00	13.99 ± 3.85	12.683	0.013	0.009	14.16 ± 3.28	14.28 ± 2.96	99,048	0.687	0.013
LSS	26.20 ± 5.39	28.10 ± 4.50	26.35 ± 5.01	26.02 ± 5.28	25.50 ± 5.26	25.56 ± 6.49	29.336	< 0.001	0.027	26.15 ± 5.52	26.23 ± 5.32	99,356	0.747	0.011
PS	16.55 ± 3.18	16.23 ± 3.21	17.44 ± 2.54	16.44 ± 2.79	16.38 ± 3.46	16.51 ± 3.46	15.284	0.004	0.012	16.19 ± 3.54	16.75 ± 2.95	93,033	0.052	0.064
PL	41.93 ± 8.14	40.73 ± 8.43	44.04 ± 6.63	41.58 ± 7.25	41.47 ± 8.74	42.64 ± 8.60	15.753	0.003	0.013	41.99 ± 8.64	41.89 ± 7.85	102,208	0.691	0.013
ASBD	58.48 ± 10.70	56.96 ± 11.14	61.49 ± 8.48	58.02 ± 9.21	57.86 ± 11.61	59.15 ± 11.46	17.131	0.002	0.014	58.18 ± 11.52	58.64 ± 10.22	98,861	0.655	0.015

Values are shown as mean ± standard deviation. Comparisons across academic years were analyzed using Kruskal-Wallis tests with 4 degrees of freedom; gender comparisons were analyzed using Mann-Whitney U tests. ε² represents the epsilon-squared effect size for academic year comparisons, while r is the effect size calculated as Z/√N for gender comparisons. Post hoc tests for academic year differences were conducted using Bonferroni-adjusted Dunn tests. For ELS and LSS, 1st-year students scored significantly higher than students in other academic years. For PS, PL, and ASBD, 2nd-year students scored significantly higher than 1st-, 3rd-, and 4th-year students

Maternal education was associated with social and total life satisfaction, as well as with push, pull, and total ASBD scores. Students whose mothers had university education generally had higher social and total life satisfaction scores and higher ASBD scores than those whose mothers had primary school education. Paternal education was associated with life satisfaction scores, with higher scores generally observed among students whose fathers had university education, but it was not associated with brain drain attitude scores. Family monthly income was associated with economic, social, and total life satisfaction, with higher income groups generally reporting higher life satisfaction, but it was not associated with push, pull, or total ASBD scores. Across these comparisons, effect sizes were small (Tables 7 and 8).

**Table 7:** Scale Scores by Parental Education Level (Kruskal-Wallis Tests with Effect Sizes)

Scale	Mother’s Education Level							Father’s Education Level						
	Primary school	Secondary school	High school	University	H	*p*	ε²	Primary school	Secondary school	High school	University	H	*p*	ε²
ELS	11.67 ± 2.84	12.01 ± 3.13	11.98 ± 2.79	12.27 ± 3.07	5.398	0.145	0.003	11.80 ± 2.90	11.20 ± 2.84	11.71 ± 2.96	12.37 ± 2.91	18.130	< 0.001*	0.016
SLS	13.79 ± 3.22	14.56 ± 3.11	14.24 ± 2.78	14.58 ± 3.10	12.301	0.006*	0.010	14.05 ± 2.78	13.80 ± 3.28	13.87 ± 3.22	14.62 ± 3.00	14.097	0.003*	0.012
LSS	25.46 ± 5.46	26.57 ± 5.73	26.22 ± 4.96	26.85 ± 5.44	11.590	0.009*	0.009	25.85 ± 4.92	25.00 ± 5.55	25.58 ± 5.60	27.00 ± 5.28	19.323	< 0.001*	0.018
PS	16.03 ± 3.35	16.40 ± 3.34	16.87 ± 2.76	16.90 ± 3.22	14.190	0.003*	0.012	16.03 ± 3.50	16.67 ± 2.77	16.70 ± 3.14	16.61 ± 3.18	4.670	0.198	0.002
PL	40.85 ± 8.54	42.25 ± 8.32	42.24 ± 7.37	42.71 ± 8.19	9.639	0.022*	0.007	40.42 ± 8.53	42.17 ± 7.98	42.44 ± 8.29	42.12 ± 7.92	5.570	0.135	0.003
ASBD	56.88 ± 11.29	58.65 ± 11.12	59.11 ± 9.40	59.61 ± 10.78	11.283	0.010*	0.009	56.45 ± 11.40	58.84 ± 10.06	59.14 ± 10.87	58.73 ± 10.46	5.758	0.124	0.003

Values are shown as mean ± standard deviation. Comparisons based on mother’s and father’s education levels were analyzed using Kruskal-Wallis tests. ε² = epsilon-squared effect size. Post hoc comparisons revealed that, for mother’s education, the Primary school group scored significantly lower than the University group for SLS, LSS, PS, PL, and ASBD (all *p* < .05). For father’s education, the Secondary school group scored significantly lower than the University group for ELS (*p* < .001), the High school group scored significantly lower than the University group for SLS (*p* = .008), and both the Secondary school and High school groups scored significantly lower than the University group for LSS (both *p* < .01). *p* < .05 indicates statistical significance

**Table 8:** Scale Scores by Family Monthly Income (Kruskal-Wallis Tests with Effect Sizes)

Scale	Low	Middle	High	*H*	*p*	*ε²*
ELS	11.63 ± 2.94	12.49 ± 2.71	13.15 ± 3.50	21.556	<.001*	.021
SLS	14.04 ± 3.19	14.72 ± 2.77	13.87 ± 3.05	11.200	.004*	.010
LSS	25.67 ± 5.51	27.21 ± 4.86	27.02 ± 5.93	16.159	<.001*	.015
PS	16.49 ± 3.12	16.70 ± 3.04	16.33 ± 4.58	1.326	.515	.000
PL	42.08 ± 8.02	41.55 ± 8.04	42.26 ± 10.20	3.681	.159	.002
ASBD	58.57 ± 10.51	58.25 ± 10.50	58.59 ± 14.11	2.843	.241	.001

Values are expressed as mean ± standard deviation. ε² = epsilon-squared effect size. *p* < .05 signifies statistical significance. Post hoc comparisons based on family monthly income revealed that the Low-income group scored lower than the Middle- and High-income groups for ELS and LSS (both *p* < .01), and lower than the Middle-income group for SLS (*p* = .008)

All life satisfaction dimensions were negatively associated with brain drain attitude scores. Although these correlations were statistically significant, they were small in magnitude. The largest correlation was observed between economic life satisfaction and total ASBD score (rho = -0.291, 95% CI: -0.35 to -0.23). Williams’ test showed that this association was significantly stronger than the association between social life satisfaction and total ASBD score (t = -2.96, *p* = .003), supporting H2 (Table 9).

**Table 9:** Spearman Correlations Between Life Satisfaction and Brain Drain Scale Scores (*N* = 935)

	PS	PL	ASBD	PS [95% CI]	PL [95% CI]	ASBD [95% CI]
*ELS*	–0.260***	–0.285***	–0.291***	[–0.32, –0.20]	[–0.34, –0.23]	[–0.35, –0.23]
*SLS*	–0.176***	–0.200***	–0.205***	[–0.24, –0.11]	[–0.26, –0.14]	[–0.27, –0.14]
*LSS*	–0.243***	–0.273***	–0.278***	[–0.30, –0.18]	[–0.33, –0.21]	[–0.34, –0.22]

*** p < .001. Values are Spearman’s rho. *CI *confidence interval

In the hierarchical regression analysis, demographic variables explained a small proportion of the variance in ASBD scores (R² = 0.041). The addition of family background variables produced a modest increase (ΔR² = 0.017), while the inclusion of total LSS score in the final block provided the largest incremental contribution (ΔR² = 0.056). The final model explained 11.4% of the variance in ASBD scores (R² = 0.114; adjusted R² = 0.093) (Table 10).

**Table 10:** Hierarchical Multiple Linear Regression Predicting Brain Drain Attitudes (ASBD Total Score)

Model	*R*²	Adj. *R*²	ΔR²	F change	*p*
Block 1: Demographics	0.041	0.028	0.041	3.057	< 0.001
Block 2: Family Background	0.058	0.037	0.017	2.035	0.040
Block 3: Life Satisfaction	0.114	0.093	0.056	57.488	< 0.001

Note. Block 1 included demographic variables (gender, academic year, and age), Block 2 added family background variables (mother’s education, father’s education, and family monthly income), and Block 3 added the total Life Satisfaction Scale (LSS) score. ΔR² = change in explained variance

Among the variables retained in the final regression model, total LSS score showed the strongest independent association with ASBD score (β = -0.248). This association was negative, indicating that higher life satisfaction was independently associated with lower brain drain attitudes. Higher ASBD scores were also observed among 2nd-year students compared with 1st-year students, students aged 24 years compared with those aged 18 years, and students whose mothers had high school or university education compared with primary school education. Other covariates, including gender, paternal education, and family monthly income, did not show independent associations in the final model (Table 11).

**Table 11:** Final Hierarchical Regression Model Predicting Brain Drain Attitudes (ASBD Total Score)

Predictor	B	SE	β	t	*p*
(Intercept)	67.193	2.341	—	28.709	< 0.001
Gender (Female)	0.349	0.704	0.033	0.495	0.621
2nd Year (vs. 1st Year)	2.825	1.335	0.264	2.116	0.035
3rd Year (vs. 1st Year)	–1.028	1.644	–0.096	–0.625	0.532
4th Year (vs. 1st Year)	–2.155	1.733	–0.201	–1.243	0.214
5th Year (vs. 1st Year)	–1.713	1.965	–0.160	–0.872	0.383
Age 24 (vs. 18)	6.755	2.466	0.631	2.739	0.006
Mother Education: High School	2.612	0.984	0.244	2.656	0.008
Mother Education: University	3.564	1.120	0.333	3.181	0.002
LSS Total Score	–0.492	0.065	–0.248	–7.582	< 0.001

Only significant predictors and key reference comparisons are shown. Full model: F(22, 912) = 5.334, *p* < .001, R² = 0.114, adjusted R² = 0.093. B = unstandardized coefficient; β = standardized coefficient; SE = standard error. Variance inflation factor values ranged from 1.03 to 1.36, indicating no evidence of problematic multicollinearity

Sensitivity analysis modeling age as a continuous variable yielded findings consistent with the categorical age model, indicating that the main associations, particularly the association between LSS and ASBD scores, were not materially dependent on age coding. Supplementary logistic regression using intention to work abroad as a binary outcome also supported the relevance of life satisfaction; each one-unit increase in LSS score was associated with lower odds of reporting an intention to work abroad (OR = 0.934, *p* < .001).

## Discussion

The findings partially supported the study hypotheses. Lower life satisfaction was associated with stronger brain drain attitudes, with the economic dimension showing the largest association. Academic year, age, and selected family background variables were also related to migration-related attitudes, whereas gender showed a limited role. The final regression model identified significant independent associations between ASBD scores and life satisfaction, academic year, age, and maternal education. This pattern supports the view that brain drain attitudes among dental students are multidimensional and cannot be reduced to a single psychosocial or sociodemographic factor.

A substantial proportion of participating dental students reported an intention to work abroad after graduation (61.9%), suggesting that migration-related attitudes were common in this sample. This finding is consistent with previous studies among dental students in Türkiye and broader evidence showing high migration intentions among health professional students [10, 21, 22]. However, the observed pattern extended beyond work-abroad intention alone. Strong family support, the relatively high desire to return after migration, and the persistence of national indebtedness in approximately half of the sample suggest that these attitudes are multidimensional rather than purely exit-oriented. This is consistent with research showing that migration intentions are shaped by career preferences, family support, social ties, sense of belonging, and return plans [23, 24]. Nevertheless, migration intention should not be interpreted as equivalent to actual migration, as the transition from intention to mobility also depends on language skills, credential recognition, mobility resources, and available opportunities [25].

Life satisfaction formed one important component of this multidimensional pattern. All life satisfaction dimensions were negatively associated with brain drain attitudes, but the associations were small in magnitude. The largest correlation was observed between economic life satisfaction and total ASBD score (rho = -0.291), and Williams’ test confirmed that this association was stronger than the association between social life satisfaction and ASBD score. This supports the hypothesis that the economic dimension of well-being is more closely related to brain drain attitudes than the social dimension. This finding is consistent with previous work suggesting that life satisfaction is linked to migration-related attitudes through broader psychosocial conditions, including financial anxiety, self-efficacy, future uncertainty, hopelessness, and career stress [11, 26]. However, the strength of these associations indicates that life satisfaction should be interpreted as one relevant correlate within a broader set of migration-related influences.

Academic year showed a clearer pattern than gender. Second-year students reported the highest levels of work-abroad intention, family support, and brain drain attitude scores, whereas first-year students had higher economic and total life satisfaction. In later academic years, students generally reported lower perceived professional value and lower optimism about the future of dentistry. One possible interpretation is that the early-to-mid phase of dental training represents a period in which professional expectations are still developing, while students become increasingly aware of the demands and uncertainties of the profession. However, because this study was cross-sectional, academic-year differences should be interpreted as cohort differences rather than within-person changes over time. Previous studies also suggest that migration-related attitudes may vary across educational stages, although the direction and timing of these patterns differ between contexts [22, 27, 28].

Gender showed a more limited role, suggesting that migration-related attitudes in this sample were more closely associated with academic year, professional outlook, and family background than with gender alone. Family background variables showed selective rather than uniform associations with the outcomes: maternal education was related to several migration-related perceptions and brain drain attitude dimensions, whereas paternal education and family income were more consistently associated with life satisfaction domains than with total brain drain attitudes. This pattern suggests that family background may be associated with migration-related attitudes through different pathways rather than through a single socioeconomic gradient. In addition, family support for working abroad increased with higher parental education and family income, indicating that migration-related attitudes may be shaped not only by individual preferences but also by family resources, expectations, and perceived access to international opportunities. This interpretation is consistent with previous research showing that migration intentions are associated with financial constraints, overseas networks, language skills, and family and social ties in both migration and return decisions [3, 29].

The pattern observed for maternal education is noteworthy but should be interpreted cautiously. Students whose mothers had higher education levels showed stronger brain drain attitudes, which may reflect broader family-linked expectations, cultural capital, and awareness of international opportunities [30, 31]. However, given the small effect sizes and the cross-sectional design, these findings should be considered context-dependent rather than definitive.

The multivariable model further supports the interpretation that brain drain attitudes are shaped by several interrelated factors. Demographic variables accounted for an initial portion of the variance, family background added further explanatory value, and life satisfaction provided the largest incremental contribution. In the final model, total LSS showed the strongest independent association with ASBD scores (β = -0.248), followed by maternal education, suggesting that both subjective well-being and family-linked resources contribute to migration-related attitudes. Supplementary logistic regression using intention to work abroad as a binary outcome was consistent with this pattern, showing that higher life satisfaction was associated with lower odds of reporting an intention to work abroad. These findings are consistent with existing research suggesting that migration-related attitudes are shaped by subjective well-being, opportunity awareness, social networks, job satisfaction, working conditions, financial anxiety, and career stress [2, 3].

These findings should also be interpreted within the broader structural context of dental education and workforce planning in Türkiye. Recent professional and national data indicate a mismatch between the rapid growth in dental training capacity and the system’s ability to integrate graduates into stable and satisfying career pathways [6–8]. Although these structural factors were not directly measured in the present study, they provide important contextual background. Accordingly, the findings highlight the need to consider psychosocial, educational, family-related, and structural factors together when interpreting brain drain attitudes among dental students.

This study has several limitations. First, the use of non-probability convenience sampling and voluntary online participation may have introduced self-selection bias. Although the survey was disseminated nationally through broad student networks, including student groups and professional student associations across multiple dental faculties in Türkiye, the sampling approach did not allow confirmation of a nationally representative distribution. Therefore, the findings should be interpreted with caution and may not be generalizable to all dental students in Türkiye. Second, the cross-sectional design prevents causal inference and does not allow academic-year differences to be interpreted as within-person changes over time. Third, duplicate submissions could not be formally verified because no personally identifiable information or IP addresses were collected. Fourth, although validated instruments were used, reliability coefficients were estimated using available subscale scores, which may be less precise than item-level reliability estimates. Finally, the model included selected psychosocial, educational, and family-related variables; future studies should incorporate additional professional, institutional, and structural factors that may further clarify brain drain attitudes among dental students.

## Conclusion

Brain drain attitudes were common among participating dental students in Türkiye and were associated with subjective well-being, academic year, age, and family background. The economic dimension of life satisfaction showed the strongest negative association with brain drain attitudes. In the multivariable model, total life satisfaction, maternal education, academic year, and age remained independently associated with ASBD scores.

These findings suggest that brain drain attitudes in dental education are not merely a response to current dissatisfaction, but rather reflect a multidimensional pattern shaped by personal well-being, educational progression, family-linked resources, and broader professional conditions. Future longitudinal and nationally representative studies are needed to clarify how structural and institutional factors influence these attitudes over time. Efforts to address brain drain in dentistry may therefore need to consider both the psychosocial well-being of students and wider improvements in the professional workforce environment.

## Supplementary Information

Supplementary Material 1.

## Data Availability

The datasets used and/or analysed during the current study are available from the corresponding author on reasonable request.
