# Supplementary material for: Brain drain attitudes and life satisfaction among dental students in Türkiye: a multidimensional analysis
Source: BMC Med Educ. 2026 Jun 24;26:1157. doi: 10.1186/s12909-026-09695-6 (PMC13374179; doi:10.1186/s12909-026-09695-6)
Supplement: Supplementary file 1 — Supplementary Material 1. [file 12909_2026_9695_MOESM1_ESM.docx]

**Additional file 1**

**Supplementary Statistical Analyses**

Brain Drain Attitudes and Life Satisfaction Among Dental Students in Türkiye: A Multidimensional Analysis

N = 935

# Overview

This supplementary file presents additional statistical analyses conducted in response to reviewer comments. The analyses include: (1) a sensitivity analysis comparing categorical and continuous age coding, (2) a formal comparison of the associations between economic and social life satisfaction and brain drain attitudes using Williams' test for dependent correlations, and (3) a supplementary binary logistic regression model examining intention to work abroad.

## Table S1. Sensitivity Analysis Comparing Categorical and Continuous Age Models

The same set of independent variables was used to compare the categorical age model and the continuous age model. Model performance was evaluated using R², adjusted R², AIC, and BIC.

| **Model** | **R²** | **Adjusted R²** | **AIC** | **BIC** |
| --- | --- | --- | --- | --- |
| Categorical age (9 dummy variables) | .114 | .093 | 7019.2 | 7135.4 |
| Continuous age | .098 | .084 | 7021.5 | 7103.8 |

**Note.** Likelihood ratio test comparing categorical and continuous age models: F(7, 912) = 2.29, p = .026. The difference in R² was small (.016), and AIC values were practically similar (Δ = 2.3). The association between life satisfaction and brain drain attitudes remained stable across age coding approaches (LSS: B = -0.496, p < .001 in the continuous age model).

## Table S2. Williams Test Comparing ELS-ASBD and SLS-ASBD Correlations

Williams' test for dependent correlations was used to formally examine whether the association between Economic Life Satisfaction (ELS) and total ASBD score was stronger than the association between Social Life Satisfaction (SLS) and total ASBD score.

| **Relationship / Test** | **Value** | **p value** | **95% CI** |
| --- | --- | --- | --- |
| rho(ELS, ASBD) | -.291 | < .001 | [-.348, -.231] |
| rho(SLS, ASBD) | -.205 | < .001 | [-.265, -.142] |
| rho(ELS, SLS) | .569 | < .001 | [.524, .611] |
| Williams' test | t(932) = -2.96 | .003 | — |

**Interpretation.** The negative association between ELS and total ASBD score was significantly stronger than the negative association between SLS and total ASBD score (Williams' t = -2.96, p = .003). This finding supports H2, indicating that the economic dimension of life satisfaction was more strongly related to brain drain attitudes than the social dimension.

## Table S3. Supplementary Binary Logistic Regression Model for Intention to Work Abroad

A supplementary binary logistic regression model was performed using intention to work abroad after graduation as the outcome variable. Results are reported as odds ratios (ORs) with 95% confidence intervals.

| **Predictor** | **OR** | **95% CI** | **p value** |
| --- | --- | --- | --- |
| Gender (female vs male) | 1.165 | [0.873, 1.553] | .300 |
| Year 2 (vs Year 1) | 1.207 | [0.708, 2.058] | .489 |
| Year 3 (vs Year 1) | 0.550 | [0.319, 0.948] | .031 |
| Year 4 (vs Year 1) | 0.282 | [0.156, 0.508] | < .001 |
| Year 5 (vs Year 1) | 0.222 | [0.105, 0.472] | < .001 |
| Age (continuous) | 1.197 | [1.054, 1.360] | .006 |
| Mother's education: secondary school (vs primary school) | 1.644 | [1.022, 2.643] | .040 |
| Mother's education: high school (vs primary school) | 1.537 | [1.030, 2.293] | .035 |
| Mother's education: university (vs primary school) | 2.329 | [1.467, 3.700] | < .001 |
| Father's education (all levels) | 0.76-0.89 | — | .26-.67 |
| Family income (all levels) | 0.92-1.57 | — | .22-.65 |
| Total LSS score | 0.934 | [0.908, 0.961] | < .001 |

**Note.** OR = odds ratio; CI = confidence interval; LSS = Life Satisfaction Scale. Statistically significant predictors at p < .05 are indicated by p values below .05.

**Interpretation.** Higher total LSS score was associated with lower odds of reporting an intention to work abroad (OR = 0.934, p < .001). Interpreted practically, each one-unit increase in LSS score corresponded to an approximately 6.6% decrease in the odds of intending to work abroad. Compared with first-year students, fourth- and fifth-year students had substantially lower odds of intending to work abroad (OR = 0.282 and OR = 0.222, respectively; both p < .001). Maternal university education was associated with higher odds of intending to work abroad (OR = 2.329, p < .001).

## Notes on Interpretation

These supplementary analyses were conducted to address reviewer comments and to examine the robustness and practical interpretation of the main findings. The primary manuscript retains the hierarchical linear regression model for total ASBD score as the main multivariable analysis, while the analyses reported here provide complementary evidence regarding age coding, the formal comparison of dependent correlations, and intention to work abroad as a binary outcome.
